# Bromido({2-[2-(diphenyl­phosphan­yl)benzyl­idene]hydrazin-1-yl­idene}(4-meth­oxy­anilino)methane­thiol­ato)palladium(II) acetone monosolvate

**DOI:** 10.1107/S1600536812028760

**Published:** 2012-06-30

**Authors:** Khalisah Asilah Mokthar, Mustaffa Shamsuddin, Mohd Mustaqim Rosli, Hoong-Kun Fun

**Affiliations:** a Department of Chemistry, Faculty of Science, Universiti Teknologi Malaysia, 81310 UTM Skudai, Johor, Malaysia; b Ibnu Sina Institute for Fundamental Science Studies, Universiti Teknologi Malaysia, 81310 UTM Skudai, Johor, Malaysia; c X-ray Crystallography Unit, School of Physics, Universiti Sains Malaysia, 11800 USM, Penang, Malaysia

## Abstract

In the title compound, [PdBr(C_27_H_23_N_3_OPS)]·C_3_H_6_O, the coordination geometry about the Pd^II^ atom is distorted square-planar, arising from the attached Br, S, P and N atoms (N and Br are *trans*), the maximum deviation from the plane being 0.2053 (4) Å for the N atom. The three benzene rings attached to the P atom make dihedral angles of 69.78 (7), 87.05 (7) and 77.50 (7)° with each other. An intra­molecular C—H⋯N hydrogen bond forms an *S*(6) ring motif. In the crystal, the complex mol­ecules form infinite chains along the *a*-axis direction through C—H⋯Br inter­actions, and a C—H⋯O inter­action links the main mol­ecule with the acetone solvent mol­ecule.

## Related literature

For the properties of palladium(II)–imino­phosphine complexes, see: Mahamo *et al.* (2012 ▶); Nobre & Monteiro (2009 ▶); Scrivanti *et al.* (2009 ▶); Sánchez *et al.* (2010 ▶); Mogorosi *et al.* (2011 ▶). For hydrogen-bond motifs, see: Bernstein *et al.* (1995 ▶).
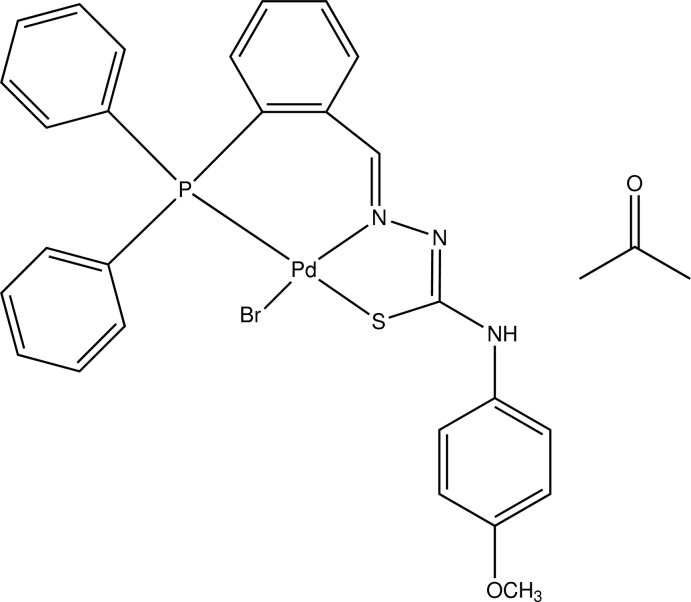


## Experimental

### 

#### Crystal data

[PdBr(C_27_H_23_N_3_OPS)]·C_3_H_6_O
*M* *_r_* = 712.90
Monoclinic, 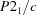

*a* = 9.7594 (1) Å
*b* = 13.7946 (2) Å
*c* = 21.8681 (3) Å
β = 104.092 (1)°
*V* = 2855.44 (6) Å^3^
*Z* = 4
Mo *K*α radiation
μ = 2.21 mm^−1^
*T* = 100 K
0.34 × 0.29 × 0.28 mm

#### Data collection

Bruker SMART APEXII CCD diffractometer
Absorption correction: multi-scan (*SADABS*; Bruker, 2009 ▶) *T*_min_ = 0.519, *T*_max_ = 0.581
39886 measured reflections
10415 independent reflections
8861 reflections with *I* > 2σ(*I*)
*R*_int_ = 0.020

#### Refinement

*R*[*F* ^2^ > 2σ(*F* ^2^)] = 0.023
*wR*(*F* ^2^) = 0.055
*S* = 1.04
10415 reflections
359 parameters
H atoms treated by a mixture of independent and constrained refinement
Δρ_max_ = 0.57 e Å^−3^
Δρ_min_ = −0.51 e Å^−3^

### 

Data collection: *APEX2* (Bruker, 2009 ▶); cell refinement: *SAINT* (Bruker, 2009 ▶); data reduction: *SAINT*; program(s) used to solve structure: *SHELXTL* (Sheldrick, 2008 ▶); program(s) used to refine structure: *SHELXTL*; molecular graphics: *SHELXTL*; software used to prepare material for publication: *SHELXTL* and *PLATON* (Spek, 2009 ▶).

## Supplementary Material

Crystal structure: contains datablock(s) I, global. DOI: 10.1107/S1600536812028760/hb6867sup1.cif

Structure factors: contains datablock(s) I. DOI: 10.1107/S1600536812028760/hb6867Isup2.hkl

Additional supplementary materials:  crystallographic information; 3D view; checkCIF report

## Figures and Tables

**Table:** 

Pd1—N1	2.0243 (12)
Pd1—P1	2.2470 (4)
Pd1—S1	2.3260 (4)
Pd1—Br1	2.4202 (2)

**Table:** 

N1—Pd1—P1	90.13 (3)
N1—Pd1—S1	83.90 (3)
P1—Pd1—S1	165.537 (15)
N1—Pd1—Br1	173.90 (3)
P1—Pd1—Br1	93.172 (10)
S1—Pd1—Br1	94.023 (10)

**Table 2:** Hydrogen-bond geometry (Å, °)

*D*—H⋯*A*	*D*—H	H⋯*A*	*D*⋯*A*	*D*—H⋯*A*
C26—H26*A*⋯N2	0.95	2.29	2.890 (2)	121
C3—H3*A*⋯Br1^i^	0.95	2.87	3.5520 (14)	129
C9—H9*A*⋯O2^ii^	0.95	2.58	3.318 (2)	135

Symmetry codes: (i) 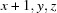; (ii) 
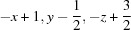
.

**Fig. 1.**
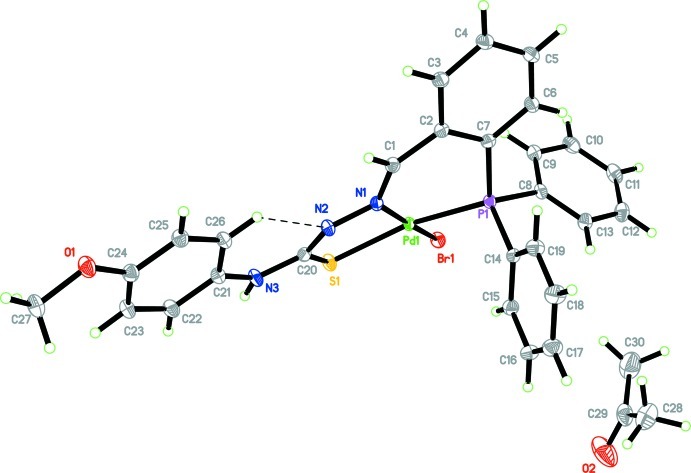
The molecular structure, showing 50% probability displacement ellipsoids. Hydrogen atoms are shown as spheres of arbitrary radius.

**Fig. 2.**
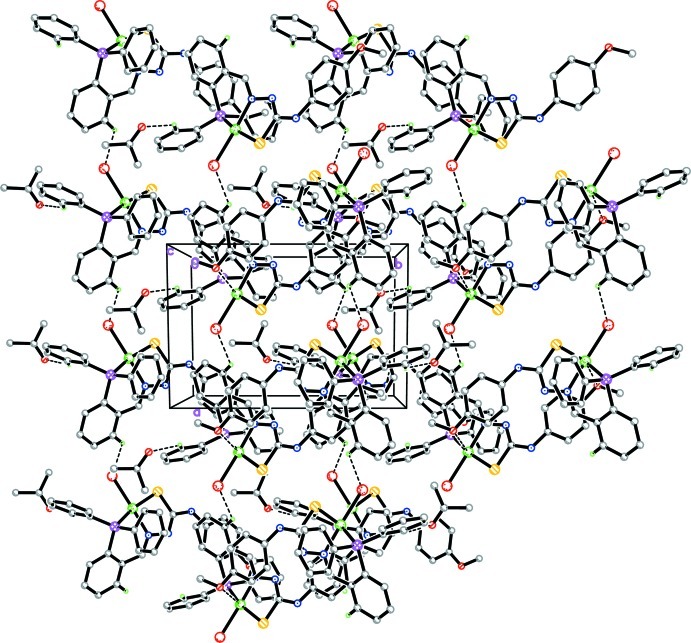
The crystal packing of (I). Dashed lines indicate hydrogen bonds. H atoms not involved in the hydrogen bond interactions have been omitted for clarity.

## supplementary crystallographic information

### Comment

Iminophosphines containing heteroatoms such as nitrogen, oxygen or sulfur have attracted considerable attention with the expectation that the functionality could participate in the chemistry. These functionalized ligands may act as chelating ligands, using phosphorus and other atom(*s*), for example, the nitrogen, oxygen or sulfur as donor atoms. Interest in the metal complexes of these hemilabile ligands and their applications in catalysis have been steadily growing as the different features associated with each donor atom offer properties unique to their metal. In particular, the palladium(II)-iminophosphine complexes have shown great potential as catalysts for the formation of new C—C bonds in organic synthesis (Mahamo *et al.*, 2012, Nobre *et al.*, 2009, Scrivanti *et al.*, 2009, Sánchez *et al.*, 2010) and in oligomerization reactions (Mogorosi *et al.*, 2011).

All parameters in (I), are within normal ranges. The coordination environment around Pd^II^ atom is in a distorted square-planar configuration, formed by Br1, S1, P1 and N1 atoms with maximum deviation from the plane form by these atoms being 0.2053 (4) Å for the N1 atom. The coordination bond distances between Pd^II^ with Br1, S1, P1 and N1 atoms are 2.42015 (17), 2.3260 (4), 2.2470 (4) and 2.0243 (12) Å, respectively with angles 93.17 (1)° for Br1—Pd1—P1, 94.02 (1)° for Br1—Pd1—S1, 83.9 (3)° for S1—Pd1—N1 and 90.13 (3) for P1—Pd1—N1. The three benzene rings attached to the P1 atom make dihedral angles of 69.78 (7)° (C2—C7 and C8—C13), 87.05 (7)° (C2—C7 and C14—C19) and 77.50 (7)° (C8—C13 and C14—C19). These three benzene rings make dihedral angles of 21.99 (7)°, 86.06 (7)° and 75.78 (7)° with the C21—C26 benzene ring.

In the molecule, an intramolecular hydrogen bond of C26—H26A···N2 (Table 1) forms an S(6) hydrogen ring motif (Bernstein *et al.*, 1995).In the crystal structure, the main molecules form infinite chains along the *a*-direction through the intermolecular interaction of C3—H3A···Br1^i^ whereas the C9—H9A···O2^ii^ bond links the main molecule with the acetone (Fig. 2).

### Experimental

The title complex was prepared by a metathesis reaction of the chlorido({2-[2-(diphenylphosphanyl)benzylidene]hydrazin-1-ylidene}(4-methoxyanilino)methanethiolato)palladium(II) complex. Potassium bromide (70 mg, 0.57 mmol) was added to a solution of the chloropalladium(II) complex (30 mg, 0.06 mmol) in acetone (6 ml). The reaction mixture was allowed to stand at room temperature for 20 h. Orange blocks of the title compound, which precipitated out were filtered off, washed with cold methanol and dried *in vacuo* (yield: 80%). Melting point: 157–159 °C.

### Refinement

N bound H atom were located from a difference Fourier map and freely refined. The remaining H atoms were positioned geometrically and refined using a riding model with C—H = 0.95–0.98 Å and *U*_iso_(H) = 1.2*U*_eq_(C) or 1.5*U*_eq_(C) for methyl H atoms. A rotating group model was applied to the methyl group.

### Crystal data

**Table:**

[PdBr(C_27_H_23_N_3_OPS)]·C_3_H_6_O	*F*(000) = 1432
*M**_r_* = 712.90	*D*_x_ = 1.658 Mg m^−^^3^
Monoclinic, *P*2_1_/*c*	Mo *K*α radiation, λ = 0.71073 Å
Hall symbol: -P 2ybc	Cell parameters from 9855 reflections
*a* = 9.7594 (1) Å	θ = 2.6–32.7°
*b* = 13.7946 (2) Å	µ = 2.21 mm^−^^1^
*c* = 21.8681 (3) Å	*T* = 100 K
β = 104.092 (1)°	Block, orange
*V* = 2855.44 (6) Å^3^	0.34 × 0.29 × 0.28 mm
*Z* = 4	

### Data collection

**Table:**

Bruker SMART APEXII CCD diffractometer	10415 independent reflections
Radiation source: fine-focus sealed tube	8861 reflections with *I* > 2σ(*I*)
Graphite monochromator	*R*_int_ = 0.020
φ and ω scans	θ_max_ = 32.7°, θ_min_ = 1.9°
Absorption correction: multi-scan (*SADABS*; Bruker, 2009)	*h* = −14→13
*T*_min_ = 0.519, *T*_max_ = 0.581	*k* = −20→20
39886 measured reflections	*l* = −33→31

### Refinement

**Table:**

Refinement on *F*^2^	Primary atom site location: structure-invariant direct methods
Least-squares matrix: full	Secondary atom site location: difference Fourier map
*R*[*F*^2^ > 2σ(*F*^2^)] = 0.023	Hydrogen site location: inferred from neighbouring sites
*wR*(*F*^2^) = 0.055	H atoms treated by a mixture of independent and constrained refinement
*S* = 1.04	*w* = 1/[σ^2^(*F*_o_^2^) + (0.0214*P*)^2^ + 1.9887*P*] where *P* = (*F*_o_^2^ + 2*F*_c_^2^)/3
10415 reflections	(Δ/σ)_max_ = 0.003
359 parameters	Δρ_max_ = 0.57 e Å^−^^3^
0 restraints	Δρ_min_ = −0.51 e Å^−^^3^

### Special details

**Table:**

Experimental. The crystal was placed in the cold stream of an Oxford Cryosystems Cobra open-flow nitrogen cryostat (Cosier & Glazer, 1986) operating at 100.0 (1) K.
Geometry. All e.s.d.'s (except the e.s.d. in the dihedral angle between two l.s. planes) are estimated using the full covariance matrix. The cell e.s.d.'s are taken into account individually in the estimation of e.s.d.'s in distances, angles and torsion angles; correlations between e.s.d.'s in cell parameters are only used when they are defined by crystal symmetry. An approximate (isotropic) treatment of cell e.s.d.'s is used for estimating e.s.d.'s involving l.s. planes.
Refinement. Refinement of *F*^2^ against ALL reflections. The weighted *R*-factor *wR* and goodness of fit *S* are based on *F*^2^, conventional *R*-factors *R* are based on *F*, with *F* set to zero for negative *F*^2^. The threshold expression of *F*^2^ > σ(*F*^2^) is used only for calculating *R*-factors(gt) *etc*. and is not relevant to the choice of reflections for refinement. *R*-factors based on *F*^2^ are statistically about twice as large as those based on *F*, and *R*- factors based on ALL data will be even larger.

### Fractional atomic coordinates and isotropic or equivalent isotropic displacement parameters (Å^2^)

**Table:**

	*x*	*y*	*z*	*U*_iso_*/*U*_eq_	
Pd1	0.704790 (10)	0.779680 (8)	0.641136 (5)	0.01125 (3)	
Br1	0.488416 (14)	0.688667 (11)	0.631039 (7)	0.01661 (3)	
S1	0.60716 (4)	0.88536 (3)	0.559230 (18)	0.01615 (7)	
P1	0.80778 (4)	0.71221 (3)	0.734540 (17)	0.01189 (6)	
O1	1.14215 (11)	1.29985 (8)	0.48277 (6)	0.0204 (2)	
N1	0.88698 (12)	0.84952 (9)	0.64072 (5)	0.0121 (2)	
N2	0.88304 (12)	0.93600 (9)	0.60730 (6)	0.0141 (2)	
N3	0.74780 (13)	1.03604 (9)	0.53199 (6)	0.0163 (2)	
C1	1.01540 (14)	0.82094 (11)	0.66483 (7)	0.0142 (3)	
H1A	1.0873	0.8613	0.6562	0.017*	
C2	1.06536 (14)	0.73545 (10)	0.70334 (7)	0.0132 (2)	
C3	1.20598 (14)	0.70940 (11)	0.70649 (7)	0.0156 (3)	
H3A	1.2597	0.7471	0.6844	0.019*	
C4	1.26828 (15)	0.62984 (11)	0.74116 (7)	0.0175 (3)	
H4A	1.3628	0.6125	0.7417	0.021*	
C5	1.19220 (15)	0.57571 (11)	0.77498 (7)	0.0183 (3)	
H5A	1.2348	0.5216	0.7991	0.022*	
C6	1.05325 (15)	0.60090 (11)	0.77346 (7)	0.0162 (3)	
H6A	1.0020	0.5642	0.7972	0.019*	
C7	0.98815 (14)	0.67937 (10)	0.73746 (7)	0.0133 (2)	
C8	0.73152 (14)	0.60811 (11)	0.76440 (7)	0.0139 (2)	
C9	0.71690 (15)	0.52162 (11)	0.73022 (7)	0.0173 (3)	
H9A	0.7438	0.5185	0.6913	0.021*	
C10	0.66292 (16)	0.44031 (11)	0.75344 (8)	0.0198 (3)	
H10A	0.6544	0.3810	0.7307	0.024*	
C11	0.62110 (16)	0.44505 (12)	0.80996 (8)	0.0216 (3)	
H11A	0.5845	0.3890	0.8257	0.026*	
C12	0.63286 (16)	0.53128 (12)	0.84311 (8)	0.0214 (3)	
H12A	0.6029	0.5348	0.8813	0.026*	
C13	0.68869 (15)	0.61310 (11)	0.82056 (7)	0.0176 (3)	
H13A	0.6975	0.6722	0.8435	0.021*	
C14	0.81635 (14)	0.80673 (10)	0.79286 (7)	0.0134 (2)	
C15	0.69068 (15)	0.85482 (11)	0.79408 (7)	0.0170 (3)	
H15A	0.6055	0.8383	0.7644	0.020*	
C16	0.69001 (16)	0.92636 (12)	0.83842 (8)	0.0195 (3)	
H16A	0.6044	0.9584	0.8394	0.023*	
C17	0.81496 (17)	0.95120 (12)	0.88152 (8)	0.0221 (3)	
H17A	0.8146	1.0002	0.9120	0.026*	
C18	0.94000 (17)	0.90461 (12)	0.88012 (8)	0.0225 (3)	
H18A	1.0252	0.9222	0.9095	0.027*	
C19	0.94167 (15)	0.83214 (11)	0.83592 (7)	0.0182 (3)	
H19A	1.0275	0.8003	0.8351	0.022*	
C20	0.76103 (14)	0.95539 (10)	0.56883 (7)	0.0137 (2)	
C21	0.85096 (15)	1.10329 (11)	0.52262 (7)	0.0148 (3)	
C22	0.80186 (15)	1.18361 (11)	0.48507 (7)	0.0181 (3)	
H22A	0.7031	1.1919	0.4687	0.022*	
C23	0.89460 (16)	1.25220 (12)	0.47100 (7)	0.0182 (3)	
H23A	0.8592	1.3071	0.4459	0.022*	
C24	1.03915 (15)	1.23961 (11)	0.49406 (7)	0.0166 (3)	
C25	1.08861 (15)	1.15945 (11)	0.53156 (7)	0.0166 (3)	
H25A	1.1875	1.1509	0.5472	0.020*	
C26	0.99713 (15)	1.09190 (11)	0.54654 (7)	0.0155 (3)	
H26A	1.0330	1.0382	0.5728	0.019*	
C27	1.09781 (17)	1.38688 (12)	0.44904 (8)	0.0215 (3)	
H27A	1.1808	1.4238	0.4449	0.032*	
H27B	1.0425	1.4257	0.4719	0.032*	
H27C	1.0395	1.3711	0.4070	0.032*	
O2	0.28740 (16)	0.89352 (14)	0.89867 (9)	0.0568 (5)	
C28	0.5216 (2)	0.88159 (18)	0.96035 (10)	0.0389 (5)	
H28A	0.5261	0.9520	0.9552	0.058*	
H28B	0.6047	0.8514	0.9506	0.058*	
H28C	0.5200	0.8665	1.0040	0.058*	
C29	0.39116 (18)	0.84330 (15)	0.91678 (9)	0.0294 (4)	
C30	0.3951 (3)	0.74038 (17)	0.89679 (11)	0.0439 (5)	
H30A	0.2984	0.7168	0.8804	0.066*	
H30B	0.4424	0.7009	0.9330	0.066*	
H30C	0.4471	0.7358	0.8638	0.066*	
H1N3	0.666 (2)	1.0483 (14)	0.5108 (9)	0.017 (5)*	

### Atomic displacement parameters (Å^2^)

**Table:**

	*U*^11^	*U*^22^	*U*^33^	*U*^12^	*U*^13^	*U*^23^
Pd1	0.01062 (4)	0.01195 (5)	0.01123 (5)	0.00014 (3)	0.00274 (3)	0.00117 (4)
Br1	0.01181 (6)	0.01856 (7)	0.01935 (7)	−0.00098 (5)	0.00360 (5)	0.00368 (5)
S1	0.01293 (14)	0.01705 (17)	0.01712 (17)	−0.00071 (12)	0.00104 (12)	0.00493 (13)
P1	0.01176 (14)	0.01188 (16)	0.01216 (16)	−0.00036 (12)	0.00319 (12)	0.00106 (12)
O1	0.0186 (5)	0.0174 (5)	0.0254 (6)	−0.0022 (4)	0.0054 (4)	0.0058 (4)
N1	0.0139 (5)	0.0115 (5)	0.0110 (5)	−0.0003 (4)	0.0030 (4)	0.0009 (4)
N2	0.0157 (5)	0.0117 (5)	0.0148 (6)	−0.0009 (4)	0.0033 (4)	0.0027 (4)
N3	0.0141 (5)	0.0155 (6)	0.0180 (6)	0.0006 (4)	0.0013 (4)	0.0060 (5)
C1	0.0138 (6)	0.0146 (7)	0.0147 (6)	−0.0011 (5)	0.0046 (5)	0.0007 (5)
C2	0.0127 (5)	0.0138 (7)	0.0131 (6)	0.0000 (4)	0.0029 (5)	−0.0006 (5)
C3	0.0132 (6)	0.0168 (7)	0.0170 (7)	0.0001 (5)	0.0041 (5)	0.0005 (5)
C4	0.0144 (6)	0.0186 (7)	0.0197 (7)	0.0023 (5)	0.0044 (5)	0.0011 (6)
C5	0.0181 (6)	0.0169 (7)	0.0201 (7)	0.0043 (5)	0.0048 (5)	0.0036 (6)
C6	0.0163 (6)	0.0158 (7)	0.0168 (7)	0.0013 (5)	0.0046 (5)	0.0031 (5)
C7	0.0127 (5)	0.0140 (6)	0.0132 (6)	0.0002 (5)	0.0034 (5)	−0.0007 (5)
C8	0.0122 (5)	0.0148 (6)	0.0137 (6)	−0.0007 (5)	0.0014 (5)	0.0037 (5)
C9	0.0176 (6)	0.0163 (7)	0.0174 (7)	−0.0004 (5)	0.0033 (5)	0.0015 (5)
C10	0.0194 (6)	0.0141 (7)	0.0242 (8)	−0.0020 (5)	0.0018 (6)	0.0013 (6)
C11	0.0179 (6)	0.0192 (7)	0.0264 (8)	−0.0049 (5)	0.0027 (6)	0.0076 (6)
C12	0.0211 (7)	0.0246 (8)	0.0190 (7)	−0.0037 (6)	0.0060 (6)	0.0050 (6)
C13	0.0191 (6)	0.0178 (7)	0.0161 (7)	−0.0019 (5)	0.0048 (5)	0.0010 (5)
C14	0.0152 (6)	0.0130 (6)	0.0124 (6)	0.0003 (5)	0.0044 (5)	0.0008 (5)
C15	0.0163 (6)	0.0181 (7)	0.0165 (7)	0.0015 (5)	0.0039 (5)	−0.0002 (5)
C16	0.0215 (7)	0.0180 (7)	0.0204 (7)	0.0028 (5)	0.0075 (6)	−0.0020 (6)
C17	0.0252 (7)	0.0190 (8)	0.0218 (8)	0.0008 (6)	0.0052 (6)	−0.0047 (6)
C18	0.0210 (7)	0.0227 (8)	0.0212 (8)	−0.0006 (6)	0.0002 (6)	−0.0057 (6)
C19	0.0163 (6)	0.0183 (7)	0.0189 (7)	0.0007 (5)	0.0024 (5)	−0.0020 (5)
C20	0.0158 (6)	0.0129 (6)	0.0134 (6)	0.0001 (5)	0.0053 (5)	0.0001 (5)
C21	0.0160 (6)	0.0135 (6)	0.0148 (6)	0.0000 (5)	0.0032 (5)	0.0014 (5)
C22	0.0162 (6)	0.0178 (7)	0.0190 (7)	0.0008 (5)	0.0018 (5)	0.0032 (6)
C23	0.0202 (6)	0.0152 (7)	0.0184 (7)	0.0008 (5)	0.0033 (5)	0.0043 (5)
C24	0.0186 (6)	0.0155 (7)	0.0158 (7)	−0.0019 (5)	0.0046 (5)	−0.0001 (5)
C25	0.0157 (6)	0.0172 (7)	0.0162 (7)	0.0000 (5)	0.0026 (5)	0.0010 (5)
C26	0.0174 (6)	0.0133 (6)	0.0151 (6)	0.0007 (5)	0.0028 (5)	0.0024 (5)
C27	0.0247 (7)	0.0168 (7)	0.0222 (8)	−0.0033 (6)	0.0044 (6)	0.0047 (6)
O2	0.0318 (8)	0.0650 (12)	0.0747 (13)	0.0166 (8)	0.0149 (8)	0.0380 (10)
C28	0.0411 (11)	0.0494 (13)	0.0270 (10)	−0.0042 (9)	0.0097 (8)	−0.0033 (9)
C29	0.0239 (8)	0.0400 (11)	0.0266 (9)	0.0040 (7)	0.0105 (7)	0.0133 (8)
C30	0.0570 (14)	0.0432 (13)	0.0313 (11)	−0.0085 (11)	0.0107 (10)	−0.0021 (9)

### Geometric parameters (Å, º)

**Table:**

Pd1—N1	2.0243 (12)	C12—C13	1.395 (2)
Pd1—P1	2.2470 (4)	C12—H12A	0.9500
Pd1—S1	2.3260 (4)	C13—H13A	0.9500
Pd1—Br1	2.4202 (2)	C14—C19	1.394 (2)
S1—C20	1.7547 (14)	C14—C15	1.400 (2)
P1—C7	1.8037 (14)	C15—C16	1.385 (2)
P1—C8	1.8109 (15)	C15—H15A	0.9500
P1—C14	1.8115 (15)	C16—C17	1.390 (2)
O1—C24	1.3722 (18)	C16—H16A	0.9500
O1—C27	1.4203 (19)	C17—C18	1.386 (2)
N1—C1	1.2971 (17)	C17—H17A	0.9500
N1—N2	1.3945 (17)	C18—C19	1.393 (2)
N2—C20	1.3075 (18)	C18—H18A	0.9500
N3—C20	1.3610 (19)	C19—H19A	0.9500
N3—C21	1.4202 (19)	C21—C22	1.393 (2)
N3—H1N3	0.836 (19)	C21—C26	1.4035 (19)
C1—C2	1.462 (2)	C22—C23	1.394 (2)
C1—H1A	0.9500	C22—H22A	0.9500
C2—C3	1.4040 (19)	C23—C24	1.389 (2)
C2—C7	1.413 (2)	C23—H23A	0.9500
C3—C4	1.387 (2)	C24—C25	1.391 (2)
C3—H3A	0.9500	C25—C26	1.384 (2)
C4—C5	1.387 (2)	C25—H25A	0.9500
C4—H4A	0.9500	C26—H26A	0.9500
C5—C6	1.392 (2)	C27—H27A	0.9800
C5—H5A	0.9500	C27—H27B	0.9800
C6—C7	1.397 (2)	C27—H27C	0.9800
C6—H6A	0.9500	O2—C29	1.211 (2)
C8—C13	1.392 (2)	C28—C29	1.489 (3)
C8—C9	1.397 (2)	C28—H28A	0.9800
C9—C10	1.387 (2)	C28—H28B	0.9800
C9—H9A	0.9500	C28—H28C	0.9800
C10—C11	1.394 (2)	C29—C30	1.489 (3)
C10—H10A	0.9500	C30—H30A	0.9800
C11—C12	1.383 (2)	C30—H30B	0.9800
C11—H11A	0.9500	C30—H30C	0.9800
			
N1—Pd1—P1	90.13 (3)	C19—C14—C15	119.75 (14)
N1—Pd1—S1	83.90 (3)	C19—C14—P1	122.61 (11)
P1—Pd1—S1	165.537 (15)	C15—C14—P1	117.64 (11)
N1—Pd1—Br1	173.90 (3)	C16—C15—C14	120.30 (14)
P1—Pd1—Br1	93.172 (10)	C16—C15—H15A	119.9
S1—Pd1—Br1	94.023 (10)	C14—C15—H15A	119.9
C20—S1—Pd1	94.29 (5)	C15—C16—C17	119.83 (14)
C7—P1—C8	105.74 (7)	C15—C16—H16A	120.1
C7—P1—C14	106.17 (7)	C17—C16—H16A	120.1
C8—P1—C14	105.63 (7)	C18—C17—C16	120.15 (15)
C7—P1—Pd1	110.66 (5)	C18—C17—H17A	119.9
C8—P1—Pd1	121.60 (5)	C16—C17—H17A	119.9
C14—P1—Pd1	106.00 (5)	C17—C18—C19	120.47 (14)
C24—O1—C27	117.48 (12)	C17—C18—H18A	119.8
C1—N1—N2	111.94 (11)	C19—C18—H18A	119.8
C1—N1—Pd1	128.05 (10)	C18—C19—C14	119.49 (14)
N2—N1—Pd1	119.82 (8)	C18—C19—H19A	120.3
C20—N2—N1	114.64 (12)	C14—C19—H19A	120.3
C20—N3—C21	130.61 (12)	N2—C20—N3	119.24 (13)
C20—N3—H1N3	115.7 (13)	N2—C20—S1	125.67 (11)
C21—N3—H1N3	113.7 (13)	N3—C20—S1	115.09 (10)
N1—C1—C2	129.27 (13)	C22—C21—C26	118.78 (13)
N1—C1—H1A	115.4	C22—C21—N3	116.77 (13)
C2—C1—H1A	115.4	C26—C21—N3	124.40 (13)
C3—C2—C7	118.33 (13)	C21—C22—C23	121.41 (14)
C3—C2—C1	114.81 (12)	C21—C22—H22A	119.3
C7—C2—C1	126.86 (12)	C23—C22—H22A	119.3
C4—C3—C2	121.49 (14)	C24—C23—C22	119.45 (14)
C4—C3—H3A	119.3	C24—C23—H23A	120.3
C2—C3—H3A	119.3	C22—C23—H23A	120.3
C5—C4—C3	119.80 (13)	O1—C24—C23	125.66 (14)
C5—C4—H4A	120.1	O1—C24—C25	115.03 (13)
C3—C4—H4A	120.1	C23—C24—C25	119.31 (14)
C4—C5—C6	119.85 (14)	C26—C25—C24	121.57 (13)
C4—C5—H5A	120.1	C26—C25—H25A	119.2
C6—C5—H5A	120.1	C24—C25—H25A	119.2
C5—C6—C7	120.90 (14)	C25—C26—C21	119.47 (14)
C5—C6—H6A	119.6	C25—C26—H26A	120.3
C7—C6—H6A	119.6	C21—C26—H26A	120.3
C6—C7—C2	119.60 (13)	O1—C27—H27A	109.5
C6—C7—P1	121.37 (11)	O1—C27—H27B	109.5
C2—C7—P1	119.02 (11)	H27A—C27—H27B	109.5
C13—C8—C9	120.06 (14)	O1—C27—H27C	109.5
C13—C8—P1	121.13 (12)	H27A—C27—H27C	109.5
C9—C8—P1	118.81 (11)	H27B—C27—H27C	109.5
C10—C9—C8	119.56 (14)	C29—C28—H28A	109.5
C10—C9—H9A	120.2	C29—C28—H28B	109.5
C8—C9—H9A	120.2	H28A—C28—H28B	109.5
C9—C10—C11	120.39 (15)	C29—C28—H28C	109.5
C9—C10—H10A	119.8	H28A—C28—H28C	109.5
C11—C10—H10A	119.8	H28B—C28—H28C	109.5
C12—C11—C10	120.00 (14)	O2—C29—C30	121.9 (2)
C12—C11—H11A	120.0	O2—C29—C28	121.5 (2)
C10—C11—H11A	120.0	C30—C29—C28	116.55 (18)
C11—C12—C13	120.04 (15)	C29—C30—H30A	109.5
C11—C12—H12A	120.0	C29—C30—H30B	109.5
C13—C12—H12A	120.0	H30A—C30—H30B	109.5
C8—C13—C12	119.93 (15)	C29—C30—H30C	109.5
C8—C13—H13A	120.0	H30A—C30—H30C	109.5
C12—C13—H13A	120.0	H30B—C30—H30C	109.5
			
N1—Pd1—S1—C20	−8.51 (6)	Pd1—P1—C8—C9	62.03 (12)
P1—Pd1—S1—C20	57.59 (7)	C13—C8—C9—C10	−1.6 (2)
Br1—Pd1—S1—C20	177.24 (5)	P1—C8—C9—C10	177.93 (11)
N1—Pd1—P1—C7	−38.54 (6)	C8—C9—C10—C11	1.1 (2)
S1—Pd1—P1—C7	−103.92 (7)	C9—C10—C11—C12	0.2 (2)
Br1—Pd1—P1—C7	136.33 (5)	C10—C11—C12—C13	−1.1 (2)
N1—Pd1—P1—C8	−163.47 (7)	C9—C8—C13—C12	0.8 (2)
S1—Pd1—P1—C8	131.15 (7)	P1—C8—C13—C12	−178.73 (11)
Br1—Pd1—P1—C8	11.40 (6)	C11—C12—C13—C8	0.5 (2)
N1—Pd1—P1—C14	76.16 (6)	C7—P1—C14—C19	−9.13 (15)
S1—Pd1—P1—C14	10.78 (8)	C8—P1—C14—C19	102.87 (13)
Br1—Pd1—P1—C14	−108.97 (5)	Pd1—P1—C14—C19	−126.86 (12)
P1—Pd1—N1—C1	31.57 (12)	C7—P1—C14—C15	171.21 (11)
S1—Pd1—N1—C1	−161.62 (13)	C8—P1—C14—C15	−76.79 (13)
P1—Pd1—N1—N2	−153.83 (10)	Pd1—P1—C14—C15	53.48 (12)
S1—Pd1—N1—N2	12.97 (9)	C19—C14—C15—C16	−1.0 (2)
C1—N1—N2—C20	163.04 (13)	P1—C14—C15—C16	178.68 (12)
Pd1—N1—N2—C20	−12.37 (16)	C14—C15—C16—C17	0.6 (2)
N2—N1—C1—C2	179.29 (14)	C15—C16—C17—C18	0.1 (3)
Pd1—N1—C1—C2	−5.8 (2)	C16—C17—C18—C19	−0.4 (3)
N1—C1—C2—C3	162.00 (15)	C17—C18—C19—C14	0.1 (3)
N1—C1—C2—C7	−19.1 (3)	C15—C14—C19—C18	0.6 (2)
C7—C2—C3—C4	1.1 (2)	P1—C14—C19—C18	−179.03 (12)
C1—C2—C3—C4	−179.93 (14)	N1—N2—C20—N3	−177.19 (12)
C2—C3—C4—C5	−1.7 (2)	N1—N2—C20—S1	2.55 (19)
C3—C4—C5—C6	0.7 (2)	C21—N3—C20—N2	6.7 (2)
C4—C5—C6—C7	1.0 (2)	C21—N3—C20—S1	−173.09 (13)
C5—C6—C7—C2	−1.6 (2)	Pd1—S1—C20—N2	6.11 (13)
C5—C6—C7—P1	179.50 (12)	Pd1—S1—C20—N3	−174.14 (10)
C3—C2—C7—C6	0.6 (2)	C20—N3—C21—C22	−175.16 (15)
C1—C2—C7—C6	−178.32 (14)	C20—N3—C21—C26	7.3 (3)
C3—C2—C7—P1	179.52 (11)	C26—C21—C22—C23	0.0 (2)
C1—C2—C7—P1	0.6 (2)	N3—C21—C22—C23	−177.65 (14)
C8—P1—C7—C6	−16.37 (14)	C21—C22—C23—C24	1.0 (2)
C14—P1—C7—C6	95.55 (13)	C27—O1—C24—C23	5.6 (2)
Pd1—P1—C7—C6	−149.86 (11)	C27—O1—C24—C25	−175.06 (14)
C8—P1—C7—C2	164.69 (11)	C22—C23—C24—O1	178.40 (15)
C14—P1—C7—C2	−83.39 (12)	C22—C23—C24—C25	−0.9 (2)
Pd1—P1—C7—C2	31.20 (13)	O1—C24—C25—C26	−179.49 (14)
C7—P1—C8—C13	114.45 (12)	C23—C24—C25—C26	−0.1 (2)
C14—P1—C8—C13	2.16 (13)	C24—C25—C26—C21	1.1 (2)
Pd1—P1—C8—C13	−118.39 (11)	C22—C21—C26—C25	−1.0 (2)
C7—P1—C8—C9	−65.12 (12)	N3—C21—C26—C25	176.44 (14)
C14—P1—C8—C9	−177.42 (11)		

### Hydrogen-bond geometry (Å, º)

**Table:**

*D*—H···*A*	*D*—H	H···*A*	*D*···*A*	*D*—H···*A*
C26—H26*A*···N2	0.95	2.29	2.890 (2)	121
C3—H3*A*···Br1^i^	0.95	2.87	3.5520 (14)	129
C9—H9*A*···O2^ii^	0.95	2.58	3.318 (2)	135

Symmetry codes: (i) *x*+1, *y*, *z*; (ii) −*x*+1, *y*−1/2, −*z*+3/2.
